# Regulation of short-chain fatty acids in the immune system

**DOI:** 10.3389/fimmu.2023.1186892

**Published:** 2023-05-05

**Authors:** Xiao-feng Liu, Jia-hao Shao, Yi-Tao Liao, Li-Ning Wang, Yuan Jia, Peng-jun Dong, Zhi-zhong Liu, Dan-dan He, Chao Li, Xian Zhang

**Affiliations:** ^1^ Wuxi Affiliated Hospital of Nanjing University of Chinese Medicine, Wuxi, China; ^2^ School of Chinese Medicine, School of Integrated Chinese and Western Medicine, Nanjing University of Chinese Medicine, Nanjing, Jiangsu, China; ^3^ Department of Spine, Wuxi Affiliated Hospital of Nanjing University of Chinese Medicine, Wuxi, China

**Keywords:** short-chain fatty acid, innate immunity, adaptive immunity, histone deacetylase, G-protein-coupled receptor

## Abstract

A growing body of research suggests that short-chain fatty acids (SCFAs), metabolites produced by intestinal symbiotic bacteria that ferment dietary fibers (DFs), play a crucial role in the health status of symbiotes. SCFAs act on a variety of cell types to regulate important biological processes, including host metabolism, intestinal function, and immune function. SCFAs also affect the function and fate of immune cells. This finding provides a new concept in immune metabolism and a better understanding of the regulatory role of SCFAs in the immune system, which impacts the prevention and treatment of disease. The mechanism by which SCFAs induce or regulate the immune response is becoming increasingly clear. This review summarizes the different mechanisms through which SCFAs act in cells. According to the latest research, the regulatory role of SCFAs in the innate immune system, including in NLRP3 inflammasomes, receptors of TLR family members, neutrophils, macrophages, natural killer cells, eosinophils, basophils and innate lymphocyte subsets, is emphasized. The regulatory role of SCFAs in the adaptive immune system, including in T-cell subsets, B cells, and plasma cells, is also highlighted. In addition, we discuss the role that SCFAs play in regulating allergic airway inflammation, colitis, and osteoporosis by influencing the immune system. These findings provide evidence for determining treatment options based on metabolic regulation.

## Introduction

Parts of the colon and small intestine contain many microorganisms, mainly bacteria and some fungi. These microorganisms produce short-chain fatty acids (SCFAs) from dietary components in the gut and from biomolecules produced by the host (1). Intestinal SCFAs mainly include acetate (C2), propionate (C3), butyrate (C4) and valerate (C5). Most SCFAs function in the gut, but a small amount of SCFAs reach the peripheral circulation *via* the portal vein (2, 3). A growing body of evidence suggests that SCFAs regulate immunity and suppress or promote inflammatory responses in the gut and other organs (4, 5). They play an important role in the regulation of innate and adaptive immunity mediated by a variety of mechanisms, including histone deacetylase (HDAC) inhibition, G-protein-coupled receptor (GPR) signaling, acetyl-CoA production, and metabolic integration. Through a combination of these mechanisms, SCFAs induce or modulate immune responses. However, the mechanism through which SCFAs regulate the immune system is relatively complex, and the mechanism of SCFAs differs among different immune cells; thus, a comprehensive summary is currently lacking. In this review, we more comprehensively introduce the regulatory role of SCFAs in the immune system. In the innate immune system, SCFAs play a role by regulating protein molecules, including the NLRP3 inflammasome and Toll-like receptors (TLRs). SCFAs also play a role by regulating innate immune cells, including neutrophils, macrophages, natural killer cells, eosinophils, basophils, and innate lymphocyte subsets (ILCs). We also highlight the regulatory role of SCFAs in the adaptive immune system, including in T-cell subsets, B cells, and plasma cells.

## Synthesis and metabolism of SCFAs

SCFAs are the most abundant microbial metabolites in the colonic lumen and are mainly produced by the microbial fermentation of prebiotics, such as dietary fiber. Among them, the ratio of C2, C3 and C4 is approximately 3:1:1 (6). The differentiation of colon epithelial stem cells and the metabolism of facultative anaerobes in the colon ensure the anaerobic environment of the colon (7–9). Obligate anaerobes in the colon (e.g., *Clostridium* and *Bacteroides*) encode broad-spectrum enzymes that hydrolyze carbohydrates and decompose dietary fibers into sugars (10) (**Table 1**). The released sugars are then fermented through glycolysis and the pentose phosphate pathway to hydrolyze dietary fibers into SCFAs (16–18). C2 is produced by pyruvates *via* acetyl-CoA or the Wood-Ljungdahl pathway (19). C3 is synthesized from acrylates using lactic acid as a precursor and is produced by the acrylate and propylene glycol pathways or by the succinate pathway that converts succinate to methylmalonyl-CoA (20, 21). C4 is reduced by condensation of two molecules of acetyl-CoA to butyryl-CoA, which can be synthesized through the butyrate kinase and phosphotransbutyrylase pathways (22). Butyryl-CoA can also be converted to C4 *via* the acetate CoA transferase pathway (23). In addition, C4 can be synthesized from proteins *via* the lysine pathway (24). Other nutrients, including proteins and peptides, can be metabolized to produce low levels of SCFAs (1). Among them, the acidic amino acid glutamic acid mainly produces C2 and C4, and aspartic acid fermentation mainly produces C2 and C3. The deamination of the alkaline amino acids lysine, arginine and histidine produces C2 and C4. The neutral amino acid cysteine can produce C2, C3 and C4, and the main products of methionine metabolism are C3 and C4 (25). In this respect, proteins are more likely to be decomposed into small amino acids in pH neutral and weakly alkaline environments and are thus more likely to produce SCFAs in these environments (26). In short, when the pH value in the lumen is 5.5, the bacteria that produce C4 dominate; at a pH of 6.5 in the lumen, C2- and C3-producing bacteria dominate (27).

**Table 1:** SCFA Production by Microbes in the Gut.

SCFAs	Receptors that are more likely to activate	Pathways/Reactions	Producers	References
Acetate	GPR43	via acetyl-CoA	*Akkermansia muciniphila, Bacteroides* spp.*, Bifidobacterium* spp.*, Prevotella* spp.*, Ruminococcus* spp.	(11–13)
		Wood-Ljungdahl pathway	*Blautia hydrogenotrophica, Clostridium* spp.*, Streptococcus* spp.	
Propionate	GPR43 GPR41	succinate pathway	*Bacteroides* spp.*, Phascolarctobacterium succinatutens, Dialister* spp.*, Veillonella* spp.	(12–14)
		acrylate pathway	*Megasphaera elsdenii, Coprococcus catus.*	
		propanediol pathway	*Salmonella* spp.*, Roseburia inulinivorans, Ruminococcus obeum.*	
Butyrate	GPR41 GPR109A	phosphotransbutyrylase/butyrate kinase route	*Coprococcus comes, Coprococcus eutactus.*	(12–15)
		butyryl-CoA:acetate CoAtransferase rout	*Anaerostipes* spp.*(A, L), Coprococcus catus (A), Eubacterium rectale (A), Eubacterium hallii (A, L), Faecalibacterium prausnitzii (A), Roseburia* spp. *(A)*	

The concentration of SCFAs in the proximal colon was 9-131 mmol/L, while the concentration of SCFAs in the distal colon was lower (11-80 mmol/L) (2, 28). SCFAs enter cells in the following ways: the dissociated anions bind to MCT1 (SLC16A1), MCT4 (SLC16A3), SMCT1 (SLC5A8), and SMCT2 (SLC5A12)-mediated transporters and GPR receptors in a hydrogen-dependent or sodium-dependent manner (29–35). Most SCFAs are consumed by the epithelial cells of the colon (36). The remaining SCFAs enter the superior mesenteric vein, inferior mesenteric vein and portal vein through passive diffusion and active transport by transporters (C2, C3 and C4 concentrations are 262.8 μM/L, 30.3 μM/L, and 30.1 μM/L, respectively) (2, 3, 37). Some SCFAs are metabolized by the liver, and the remaining SCFAs are dispersed to peripheral circulation (the concentrations of C2, C3, and C4 were 172.9 μM/L, 3.6 μM/L, and 7.5 μM/L, respectively) (38, 39). These blood concentrations of SCFAs are thought to be high enough to affect host cells (39), depending on the type and amount of dietary fiber ingested by the host and the health status of the host (e.g., helminthic infection, viral infection, autoinflammation) (40, 41).

The receptors of SCFAs that have been widely reported are GPR41, GPR43, GPR109A, OR51E2 (human) and OLFR78 (mouse) (42–45). SCFAs with different carbon chain lengths have different abilities to activate GPR41, GPR43 and GPR109A receptors. Two to three carbon chains are more likely to activate GPR43 receptors, while 3-5 carbon chains are more likely to activate GPR41 receptors. C4 activates the GPR109A receptor more easily (43, 44). SCFAs are found in high levels in the gut, and most of these receptors are activated in intestinal tissues.

SCFAs are natural inhibitors of HDAC, of which there are 18 types (46, 47). There are four classes of HDAC as follows: Class I (HDAC1-3 and HDAC8), Class II (HDAC4-7 and HDAC9-10), Class III (SIRT1-7) and Class IV (HDAC11) (47). Different types of SCFAs have different inhibition rates of different types of HDAC. For example, C4 can inhibit HDAC up to 80%, C3 can inhibit HDAC up to 60%, and C2 has the lowest inhibition rate (48, 49). SCFAs can affect histone acetylation by regulating the homeostasis between histone acetyltransferase (HAT) and HDAC. HAT transfers acetyl groups to lysine residues in the tail, forming acetylated lysine, which neutralizes the positive charge carried by the histones (50). HDAC deacetylates the acetylated lysine in the histone tail, making the nucleosome compact and making it more difficult to perform gene transcription and expression (51, 52). Therefore, different types of SCFAs affect gene transcription in immune cells by inhibiting the activity of different types of HDACs

## Active functions and signaling pathways of SCFAs

### NF-κB signaling pathway

Nuclear factor-κB (NF-κB) mediates the transcription of various cytokines and chemokines, such as the cytokines TNF-α, TNF-β, IL-1β, IL-2, IL-3, IL-5, IL-12, and IL-18 and the chemokines IL-8, MIP-1α, MIP-2, and MCP-1 (53–56). Two subunits of NF-κB, P65 and P50, are acetylated and transferred from the cytoplasm into the nucleus to promote the secretion of proinflammatory cytokines (57). SCFAs produce anti-inflammatory effects by inhibiting NF-κB. The order of inhibition of NF-κB activity was C4>C3>C2 (58).

HDAC can regulate the secretion of inflammatory cytokines by inhibiting the acetylation of NF-KB (59). It was found that the subunits p65 and p50 of NF-κB interact with HDAC to inhibit transcription (59). Deacetylation of p65 by HDAC3 enhances the binding of p65 to IκBα, resulting in the export of the NF-κB complex from the nucleus back into the cytoplasm to inhibit the transcription of proinflammatory factors (60). C3 and C4 are known HDAC inhibitors and have been shown to regulate NF-κB activity. For example, C4 upregulates the production of IL-10 and inhibits the production of the proinflammatory molecules IL-12, TNF-α, IL-1β, and NO by inhibiting NF-κB activity (61–63).

**Figure 1:**
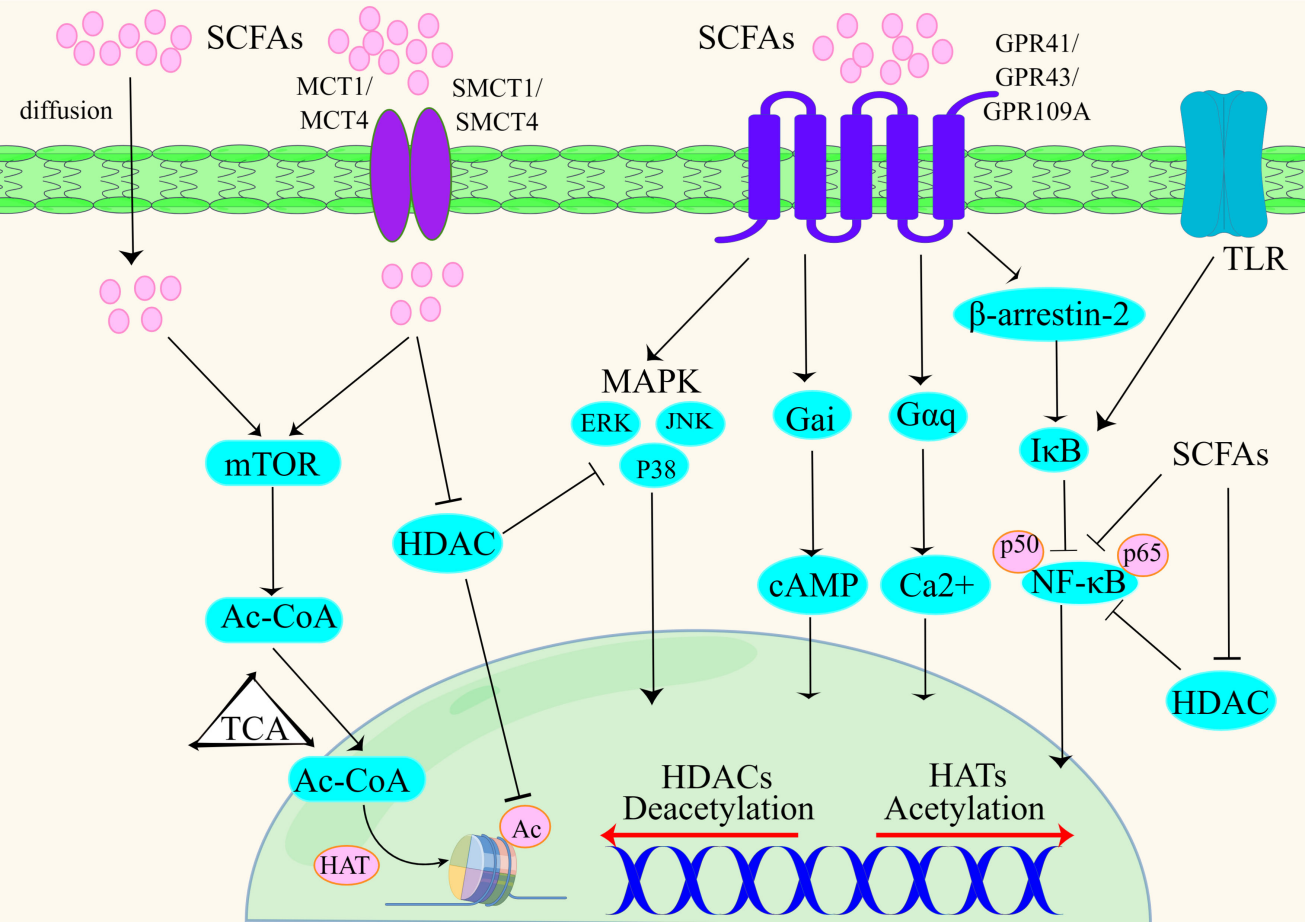
SCFAs influence the immune response by a variety of signalling pathways, including epigenetic inheritance in cells (56, 66, 67).

GPR receptors influence the secretion of inflammatory cytokines by regulating the β-arrestin 2 pathway upstream of the NF-κB signaling pathway. The GPR43 receptor reduces the level of NF-κB through the β-arrestin 2 signaling pathway and reduces the amount of the two subunits of NF-κB, p65 and p50, entering the nucleus; thus, the GPR43 receptor inhibits the transcription of proinflammatory cytokines (IL-1β and IL-6) (64, 65) (**Figure 1**).

### MAPK signaling pathway

Phosphorylated mitogen-activated protein kinase (MAPK) regulates the ERK, JNK and P38 MAPK signaling pathways, gene transcription, and proinflammatory cytokine secretion (68). The acetylated state of MAPK phosphatase-1 (MKP-1) interacts with the MAPK substrate to dephosphorylate MAPK and inhibit the activation of the ERK, JNK and P38 MAPK signaling pathways (69).

SCFAs may regulate MAPK pathways by inhibiting HDAC. HDAC1-3 deacetylates MP-1, and the deacetylation of MP-1 leads to an increase in MAPK signaling and proinflammatory cytokine secretion (70). However, the effect of HDAC may also be independent of MAPK signaling pathways. Treatment of bone marrow-derived macrophages exposed to lipopolysaccharide (LPS) with TSA (an HDAC inhibitor) inhibited TNF-α and IL-6 production in cells in a time- and dose-dependent manner. However, TSA did not inhibit ERK1/2 and p38 phosphorylation in macrophages (71).

SCFAs can participate in proinflammatory effects by activating GPR41 and GPR43 receptors. It has been shown that activation of the GPR41 and GPR43 receptors can induce ERK1/2 phosphorylation, while activation of GPR43 receptors can induce p38 MAPK phosphorylation (72). C2 activates the GPR41 and GPR43 receptors and their downstream ERK2/1 and MAPK signaling pathways and increases the production of proinflammatory factors and chemokines (72, 73) (**Figure 1**).

### mTOR signaling pathway

Rapamycin target (mTOR) is a serine/threonine protein kinase. There are two distinct functional complexes, mTORC1 and mTORC2, that regulate cell growth, proliferation, transcription, mRNA renewal, translation and other important processes (74). Activation of mTOR helps regulate barrier function in the gut and can influence the production of immune cells and cytokines. mTOR increases the acetyl-coA content *via* the glycolysis pathway, and excess acetyl-coA is converted to citrate *via* the tricarboxylic acid cycle (TCA cycle). Citrate, which is involved in the TCA cycle, is converted to acetyl-CoA in the nucleus *via* ATP-citrate lyase. Acetyl-CoA in the nucleus promotes the binding of acetyl groups to histones and increases the acetylation of histones, ultimately regulating gene expression and the production of cytokines such as IL-10 and TNF (75–77). SCFAs enter cells to inhibit HDAC and increase the acetylation of p70 S6 kinase and the phosphorylation of rS6, thereby regulating the mTOR pathway and increasing IL-10 cytokine production (74) (**Figure 1**).

The activated GPR41 receptor was shown to bind to intracellular Gαi, reducing the level of cAMP. The activated GPR43 receptor conjugates with intracellular Gαq and Gαi to inhibit cAMP levels. Increased intracellular cAMP levels facilitate the entry of intracellular calcium ions into the cytoplasm, a process that regulates gene transcription and translation in immune cells (43) (**Figure 1**).

## SCFAs and immune regulation

### Innate immunity

#### Regulation of the NLRP3 inflammasome by SCFAs

The inflammasome is a multiprotein complex assembled by intracytoplasmic pattern recognition receptors (PRRs) and is an important part of the innate immune system (78, 79). The NLRP3 inflammasome is responsible for the maturation and secretion of the related cytokines IL-1β and IL-18 (80). Studies have shown that SCFAs, after binding with GPR43 and GPR109A in intestinal epithelial cells (IECs), cause intracellular potassium ion outflow and hyperpolarization, calcium ion inflow and activation of the NLRP3 inflammasome (81). In addition, after activating the GPR43 receptor of IEC cells, SCFAs activate the NLRP3 inflammasome by increasing the phosphorylation of ERK (82). However, SCFAs have been shown to inhibit NLRP3 inflammasome activation in other cells. For example, intervention by SCFAs significantly reduced NLRP3 inflammasome activation in astrocytes (83). C4 exerts anti-inflammatory effects by inhibiting the formation and activation of the NLRP3 inflammasome in vascular endothelial cells, but C2 and C3 do not show the same effect; thus, C4 plays an anti-inflammatory role and contributes to the formation of new carotid intima (84). The results discussed above indicate that not only do the same type of SCFAs have different inhibitory or promoting effects on different types of cells, but different types of SCFAs also have different effects on the same types of cells. This reminds us of previous findings that showed that SCFAs have proinflammatory effects on some cell types, such as macrophages and microglia, and anti-inflammatory effects on others (85, 86). Therefore, how SCFAs exert their proinflammatory and anti-inflammatory effects requires further study.

#### Regulation of TLR family members by SCFAs

The expression of PRRs enables the immune system to distinguish intestinal commensal microorganisms from harmful microorganisms. TLRs, a subtype of PRRs, play an important role in the innate immune response. TLRs can promote the proliferation of intestinal epithelial cells and the expression of antimicrobial peptides (AMPs) (87). Studies have shown that C3 and C4 regulate the response of multiple TLRs and TNF-α by inhibiting the histone acetylation of HDAC (88). Among them, TLR5 is highly expressed in the colon and can recognize the flagellin of gram-negative bacteria by activating a series of pathways within the cell (89). In patients with ulcerative colitis, the concentration of SCFAs in the colon is generally consistent with the expression of TLR5 in the colon. The content of SCFAs decreases gradually from the proximal end of the colon to the distal end, and the expression of TLR5 also decreases gradually from the proximal end of the colon to the distal end, indicating that there may be a certain relationship between the two (90). Further studies showed that the regulation of TLR5 by C4 occurred at the transcriptional level rather than at the translational level. C4 activates PKC isoforms to dephosphorylate and acetylate specific protein 1 (Sp1) by serine and threonine phosphatases, respectively, and phosphorylates specific protein 3 (Sp3) by ERK-MAPK. This leads to displacement of Sp1 from the promoter and binding to Sp3, which activates the transcription of TLR5 in intestinal epithelial cells (91). This is consistent with a recent study showing that enterobacterial flagellin activates the release of anti-inflammatory factors (IL-10, TGF-β) and reduces inflammation in IECs. C4 is the main metabolite secreted by Enterobacterium, which can initiate TLR5 transcription through Sp3, upregulate TLR5 expression, and inhibit the expression and release of inflammatory factors (IL-6, IFN-γ and TNF-α) (92). In addition, TLR4 can activate innate immune responses by sensing LPS in the cell walls of gram-negative bacteria (93). C4 can promote TLR4 expression and the phosphorylation of MAPKs and NF-κB to regulate the innate immunity of colon cancer cells, but the specific mechanism remains unclear (94). To date, there are relatively few studies on the pathway mechanism of SCFA-TLRs in innate immunity, and the correlation between SCFAs and TLR signaling pathways is not clear. However, existing studies have clearly shown that SCFAs play an anti-inflammatory role by regulating the expression of TLRs, which is important for the regulation of immune homeostasis in the body.

#### Regulation of neutrophils by SCFAs

Neutrophils are considered the most abundant innate immune cells in the bone marrow and peripheral blood (95). SCFAs affect the recruitment of neutrophils to the site of inflammation and reduce inflammation. SCFAs increase the expression of L-selectin on the surface of neutrophil granulocytes, activate neutrophil chemotactic recruitment to the inflammatory site, and aggravate the inflammatory process without affecting the expression of β2 integrin mRNA (96). SCFAs induce the aggregation of neutrophils to the site of inflammation through the CPR43 receptor. This process is associated with the activation of intracellular protein kinase P38 and the coupling of Gi/o and Gq proteins (97). In a model of gout induced by sodium urate monohydrate (MSU), C2 promoted neutrophil reactive oxygen species (ROS) production in a GPR43-dependent manner, indirectly activated the NLRP3 inflammasome, led to neutrophil recruitment to the inflammatory site, promoted inflammasome activation, and promoted the release of IL-1β and IL-10 (98). C4 significantly inhibits the production of proinflammatory cytokines (e.g., IL-6, TNF-α and IFN-γ) and chemokines (e.g., CCL3, CCL4, CXCL1 and IL-8) secreted by neutrophils in the intestines of patients with colitis to reduce intestinal inflammation, and C4 inhibits the secretion of proinflammatory cytokines in an HCDAC-dependent manner (99). C2 also reduces the infiltration of pancreatic neutrophils and significantly reduces pancreatitis in mice (100).

#### Macrophages

Macrophages are essential for maintaining homeostasis in the gut (101). Previous studies have shown that the process by which C4 inhibits the production of inflammatory cytokines by intestinal macrophages is related to the inhibition of HDAC activity (102). C4 induces anti-inflammatory properties of macrophages in a GPR109-dependent manner (44). A recent study showed that C4 alters macrophage metabolism and increases their antibacterial activity. Metabolomic analysis of butyrate-treated macrophages revealed a substantial reduction in glycolysis. This was associated with higher amounts of adenosine monophosphate, an inducer of MAPK, which, in turn, inhibits mTOR. As mTOR is a positive regulator of glycolytic enzymes, its inhibition may explain the observed reduction in glycolysis (103–105). Moreover, mTOR is considered a key regulator of autophagy (106). The bacteria-associated autophagy protein microtubule-associated protein 1 light chain 3α (LC3) is a key participant in autophagy, and experiments have shown that the conversion rate of LC3 and ROS production are increased. Further analysis by single-cell RNA sequencing revealed that the C4-induced antibacterial signature is characterized by increased expression of the S100A8 and S100A9 genes, which encode calprotectin, a protein with antibacterial properties. Therefore, C4 helps increase the antibacterial activity of macrophages by inhibiting mTOR (103–105). In addition, in the presence of C4 and pertussis toxin (GPCR inhibitors), macrophages exhibit enhanced antibacterial activity, indicating that C4 enhances the antibacterial activity of macrophages without the involvement of GPCR. Further studies have shown that butyrate increases the expression of the S100A8 mRNA gene through its inhibition of HDAC3. Changes in metabolism enhance the bactericidal function of macrophages (103–105). Similarly, in mouse pancreatitis caused by *Staphylococcus aureus*, C4 enhances the antibacterial program of macrophages by inhibiting HDAC3 (107). Earlier studies have also found that the *in vitro* stimulation of mouse macrophages with butyrate leads to inhibition of inflammatory responses and decreases in nitric oxide levels, a process mediated by HDAC (102). In addition, SCFAs downregulate M2 polarization in human and mouse alveolar macrophages *in vitro* and may activate GPR43 but not GPR41. Butyrate and propionate (but not acetate) increase H3 acetylation and inhibit M2 polarization in part through HDAC inhibition (108).

#### Natural killer cells

Natural killer cells, which are the first identified ILC subtype, can respond to virus-infected or virus-transformed cells with a variety of effector functions, primarily cell killing and proinflammatory cytokine production (109, 110). The combination of IL-12 and IL-15 activates natural killer cells and promotes metabolic changes needed for increased receptor expression and cytokine secretion (111). In IL-12/IL-15-stimulated natural killer cells, C4 inhibits the expression of the cell surface receptors TRAIL, NKp44, NKp30, and NKG2D and significantly inhibits the production of the proinflammatory cytokines TNF-α, IFN-γ, IL-22, soluble Fas ligand, granzyme A, granzyme B and perforin. C3 inhibits the expression of the receptor NKp30 and the production of the proinflammatory cytokines IFN-γ and granzyme B, but C2 does not have the same effect (112). Researchers have found that C4-treated natural killer cells express higher levels of bromodomain-containing protein 2 (BRD2). BRD2 is an inflammatory cytokine that controls the production of natural killer cells (113). mTORC1 is a key regulator of natural killer cell metabolism (114). c-Myc regulates the secretion of survival cytokines in natural killer cells (115, 116). C4 has been shown to have anti-inflammatory effects by reducing mTOR activity and c-Myc mRNA expression in natural killer cells (113). In addition, C4 inhibits glycolysis and oxidative phosphorylation in natural killer cells by inhibiting the expression of the first enzyme in the glycolysis pathway, HK2 (113).

#### Eosinophils

Eosinophils promote a variety of complex immunomodulatory functions. Under inflammatory conditions, proinflammatory factors can activate eosinophils and prolong their survival. Activated eosinophils are important participants in the inflammatory process and secrete proinflammatory factors, including IL-3, IL-6, and tumor necrosis factor-α (117). These cells also secrete proinflammatory lipid mediators, including platelet activating factor and cysteine-leukotriene. Anti-inflammatory lipid mediators, including lipoxygenase, lysins and protectors, release reactive oxygen species (118–121). In a mouse model of allergic airway inflammation, a high-fiber diet, probiotics, or direct administration of SCFAs effectively reduced airway eosinophils by altering the gut microbiome and SCFA levels (122–125). Similarly, SCFAs exert the same effect in eosinophilia-related diseases (including asthma, atopic dermatitis, inflammatory bowel diseases, and eosinophilic oesophagitis) (126, 127). A recent study illustrated the mechanism through which SCFAs directly affect eosinophils. Eosinophils treated with C3 and C4 exhibit decreased cell size and number, loss of bilobate nuclei, mitochondrial membrane potential depolarization, and effector caspase activation, which results in the induction of apoptosis by regulating intracellular pathways, a process that may be mediated by inhibition of HDAC independent of the GPR41 and GPR43 receptor pathways. However, C2 does not impair the survival of eosinophils (128). These findings are consistent with a previous study that revealed that C4 alleviates airway hyperresponsiveness and eosinophilic increases in patients with allergic asthma through HDAC inhibition and a process independent of GPR41 and GPR43 receptor activation (125). C2 and C3 bind to GPR43 in human eosinophils, resulting in increased intracellular calcium influx (128). Moreover, C2 and C3 stimulate the production of ROS in human eosinophils in a concentration-dependent manner (128). Researchers have further investigated the potential of SCFAs to regulate the transcription of genes involved in eosinophilic adhesion, migration, and survival. C3 and C4 have been shown to inhibit eosinophilic adhesion and migration to the endothelial monolayer in response to eotaxin-2 (CCL24) (128). In addition, the surface expression of L-selectin on eosinophils is not affected by SCFAs (128, 129).

#### Basophils

At present, there are many studies on the relationship between SCFAs and eosinophils, but there are relatively few studies on the relationship between SCFAs and basophils. C2-treated basophils resulted in increased intracellular calcium influx, but treatment with C3 and C4 did not have the same effect (130). Activation of basophils is associated with IL-3 induction of CD69 on the cell surface (130, 131). C3 and C4 showed increased IL-3-induced CD69 expression and increased cell IL-13 and IL-4 secretion by inhibiting HDAC (130, 132, 133). In addition, C3 and C4 promote an increase in IgE-mediated basophil degranulation (130, 134). This suggests that SCFAs may be one of the important factors regulating alkaline granulocyte activation, IL-13 expression and degranulation.

#### Differential regulation of ILC subpopulations by SCFAs

ILCs were divided into groups 1 (NK cells and non-NK cells ILC1), 2 (ILC2) and 3 (ILC3) according to their developmental and functional characteristics (135). It was found that C2, C3, and C4 triggered the P13K, AKT, and mTOR signaling pathways through the excitatory action of GPR43 receptors, thereby promoting the proliferation of intestinal ILC1s and ILC3s but inhibiting the proliferation of intestinal ILC2s (136–138). Similarly, C4 has been shown to inhibit ILC2 proliferation in allergic asthma, but this process may be related to the inhibitory effect of HDAC (125). This suggests that the effect of SCFAs on ILCs is mediated by both GPR receptors and HDAC.

**Figure 2:**
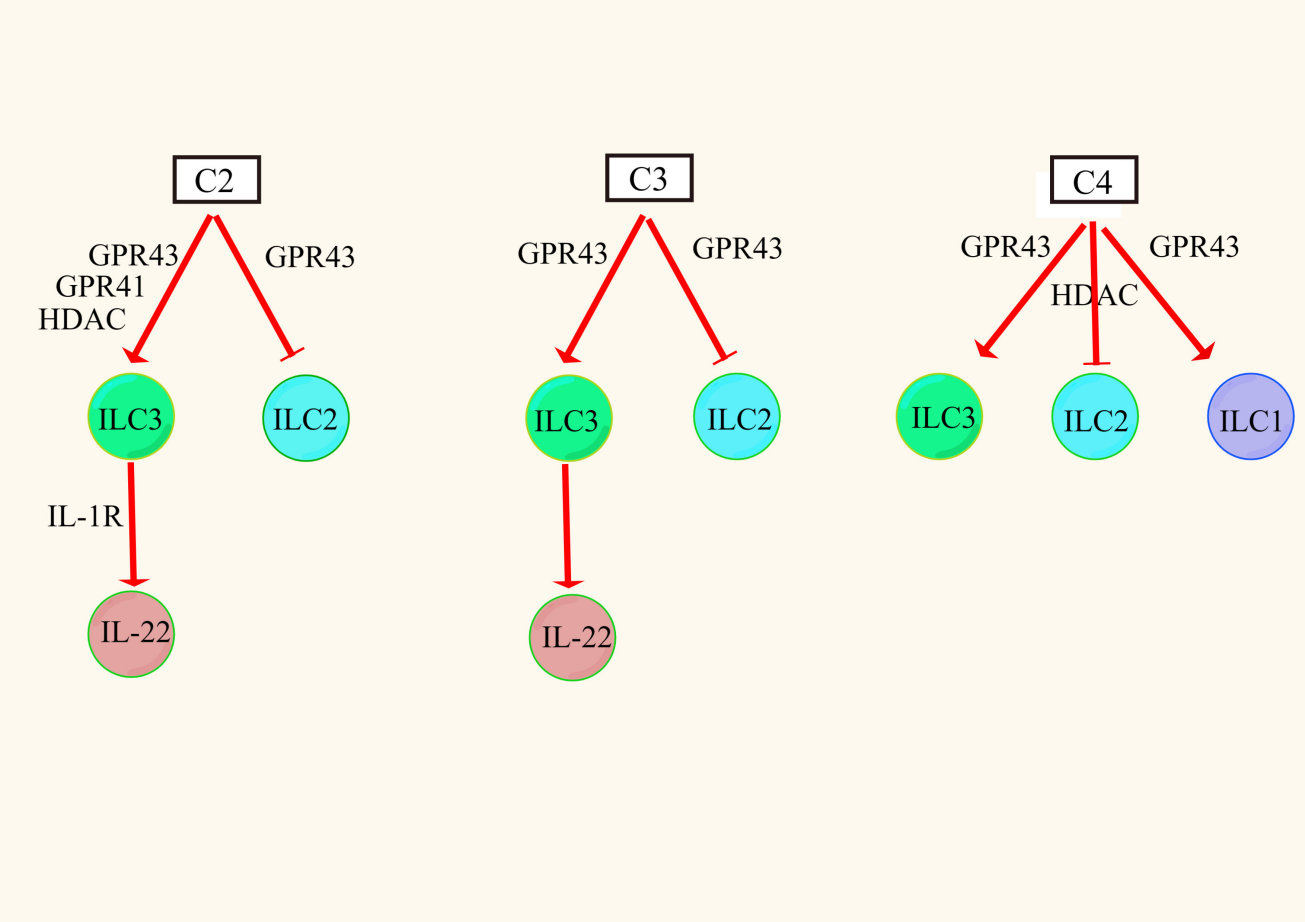
Effects of SCFAs on ILCs (125, 136–138, 140, 141).

ILC3s are a major producer of intestinal barrier IL-22, which is a member of the IL-10 family and a key cytokine regulating inflammation. It is upregulated in chronic inflammation and achieves anti-inflammatory effects by inducing intestinal epithelial cells to produce AMP and mucin and repair the integrity of the damaged intestinal epithelial barrier (139). In ILC3s, SCFAs differentially activate AKT or ERK signaling and increase IL-22 secretion *via* the AKT and STAT3 axes. Among them, C2 increased the secretion of IL-22 to a greater extent by activating the GPR43 receptor, and C3 increased the secretion of IL-22 to a lesser extent by activating the GPR43 receptor, but C4 had no effect on the secretion of IL-22 (140). The reasons for these findings may be that, on the one hand, C2 and C3 activate the GPR43 receptor in ILCs more easily than C4. On the other hand, it may be that there are other pathways of regulation. For example, C2 enhances the expression of IL-1R in ILC3 cells by activating the GPR43 receptor, and the increased level of IL-1R increases the sensitivity of IL-1β, thereby indirectly inducing the production of IL-22 (141). In addition, SCFAs can promote IL-22 secretion in ILC3s by activating the GPR41 receptor and inhibiting the HDAC pathway (**Figure 2**). After SCFAs enter cells, they upregulate the expression of hypoxia-inducing factor 1α (HIF1α) and aromatic receptor (AhR), which are differentially regulated by mTOR and STAT3 (141–143). HIF1α directly binds to the IL-22 promoter (144). Finally, histone modification increases the binding of HIF1α to the IL-22 promoter to increase IL-22 secretion (144). In conclusion, different types of SCFAs have different regulatory effects on different types of ILCs, and the mechanism is closely related to the expression of the GPR receptor and the inhibition of HDAC.

### Adaptive immunity

#### T cells

Previous studies have shown that 17 strains in the mouse gut microbiota (belonging to clusters IV, XIVa, and XVIII of *Clostridium difficile)* induce the TGF-β response by producing SCFAs, which may contribute to the differentiation and amplification of Tregs in the colons of mice (145). It was later determined that SCFAs promote the proliferation of Tregs (145). Treg cells include FoxP3^+^ T cells, which prevent inflammatory reactions in the gut by producing IL-10 (146). In the presence of Treg cell polarization, C4 treatment of naive T cells enhanced histone H3 acetylation in the promoter, specifically inducing differentiation of mouse colon Treg cells by upregulating the acetylation of conserved noncoding sequences at FoxP3 (147). SCFAs can also indirectly promote the proliferation of Treg cells and IL-10 production through other cell types. For example, C4 can act on macrophages and DCs in a GPR109A-dependent manner, indirectly inducing FoxP3^+^ T-cell and IL-10 production (44). However, a recent study found that C5 did not increase the amplification of Treg cells but increased the amount of additional acetyl-CoA in T cells, enhanced glycolysis through the mTOR activation pathway, and increased IL-10 production in lymphocytes by acting as a substrate for HAT to regulate the gene recoding process (76, 148).

SCFAs regulate T-cell metabolism through HDAC inhibition. SCFAs can increase the differentiation of naive T cells into effector T cells, such as Th1 and Th17 effector T cells, which may be related to the inhibitory activity of HDAC. In T cells, SCFAs were found to increase the acetylation of p70 S6 kinase and the phosphorylation of rS6 by inhibiting HDAC activity, thereby increasing mTOR activity to increase the production of Th17 and Th1 cells and IL-10(+) T cells (149, 150).

SCFAs can affect T cells during the antiviral process. It was found that oral SCFAs may affect CD8+ T-cell metabolism in a GPR-dependent manner and by inhibiting HDAC action during active immunity to ensure rapid activation of effector T cells in response to viral infection (151). Further studies later found that the regulation of C4 on CD8+ T cells was mediated by the inhibitory activity of HDAC, independent of the GPR41 and GPR43 receptors (152). SCFAs (specifically C4) also increase the number of CD8+ memory T cells in the spleen and liver and play an important role in establishing an optimal secondary antigen-specific response (153). This process is generally thought to increase glycolysis and mitochondrial mass to promote the survival and activation of CD8+ memory T cells (153). SCFAs have been widely recognized as an energy source for cells. In the tumor microenvironment, SCFAs can enhance the ability of CD8+ T cells to compete with tumor cells for glucose, thus increasing the survival and activation of CD8+ T cells (154). SCFAs enhance the antitumor activity of CD8+ T cells and chimeric antigen receptor (CAR) T cells through metabolic and epigenetic reprogramming (148). Therefore, SCFAs can regulate the metabolism of T cells according to the states of the host.

#### B cells

B cells require glycolysis, oxidative phosphorylation, and the synthesis of palmitic acid (PA) in the processes of proliferation, differentiation, and secretion of antibodies. Glycolysis and oxidative phosphorylation are essential for the survival of germinal B cells, and fatty acids (FAs) are involved in the process of antibody secretion by regulatory plasma cells (PCs) (155). Previous studies have shown that certain probiotics, such as *Lactobacillus and Bifidobacterium* species, increase fecal and serum IgA and IgG levels but decrease fecal and serum IgA levels in germ-free and antibiotic-treated mice (156). In mice fed prebiotics, SCFA content and IgA levels in feces and the large intestine increased in a dose-dependent manner, with increased IgA and IgG levels in serum and no change in IgE and IgM levels. These findings suggest that SCFAs produced by prebiotics may promote the differentiation of B cells and the production of antibodies (157). The differentiation of B cells into PCs and the production of antibodies require energy metabolism within the cell to produce sufficient ATP. Previous studies have shown that SCFAs can be converted to acetyl-CoA (which produces ATP in the TCA cycle) *via* acetyl/propionyl/butyryl CoA (158). Acetyl-coA is the main substrate for FA synthesis (159). FA contributes to the differentiation of plasma B cells and stimulates B cells to produce antibodies (160). The contents of acetyl-CoA, ATP, lipids, malonyl CoA and fatty acid synthase (FAS) were increased in B cells treated with SCFAs, and ATP, malonyl CoA and FAS were essential for FA production (161). In addition, SCFAs promote B-cell differentiation and antibody production by increasing glycolysis in B cells (39).

SCFAs affect B-cell differentiation and antibody production through HDAC inhibition and GPR receptor mediation. Studies have found that SCFAs change the expression of B-cell-related genes (IgGs, IgA, Igj, Igk, Igl, Aicda, Xbp1, Irf4, etc.) by inhibiting HDAC. These genes participate in the differentiation of B cells and promote their differentiation into antibody-producing PCs (39, 162–164). B cells can express the GPR receptor, and studies have found that compared with wild-type mice, mice lacking the GPR43 receptor have relatively low IgA levels in the gut (165).

SCFAs regulate B cells through a number of indirect mechanisms. SCFAs increase glucose uptake by T helper cells and follicular helper T-cell (Tfh) induction, which regulates B-cell and antibody production (39, 166). C2 amplifies TLR signals in Tfh cells, and TLR selectively changes the levels of some IgA-producing microorganisms by sensing LPS from different microorganisms (167). In addition, C2 regulates antibody secretion by regulating dendritic cells (DCs), activates B cells by producing BAFF/APRIL and produces retinoic acid (RA) to induce IgA production (165).

SCFAs can regulate the production of B10 cells (regulatory B cells (Bregs) that produce IL-10 to maintain immune homeostasis) by a different mechanism. C2 can be converted to acetyl-CoA synthetase (ACSS), which increases the differentiation of B10 cells in the peritoneal cavity of mice by promoting the acetylation of ATP and lysine produced by the TCA cycle. C3 has no direct effect on the differentiation of B10 cells (168). C4 has been reported to induce an increase in splenic B10 cells by indirectly promoting the production of the serotonin-derived metabolite 5-hydroxyindole-3-acetic acid (169). C4 can also induce IL-10-producing splenic B10 cells by regulating the circadian clock-related genes RAR-associated orphan receptor α and nuclear receptor subfamily 1 group D member 1 (170, 171). A subsequent study showed that SCFAs upregulated peritoneal B10 cells in colitis mice in a manner dependent on their HDAC inhibitory activity. Independent of the GPR receptor pathway, transcriptional analysis showed that the action of C4 on B10 cells was related to the activation of p38 MAPK (172). C5 can not only increase the secretion of IL-10 but also significantly inhibit the apoptosis of Bregs (145). These results indicate that different SCFAs have different effects on B10 cell development, which encourages us to conduct further research.

## Association of SCFAs with disease

### Allergic asthma

The pathogenesis of allergic asthma is not well understood. Clinical treatment focuses on reducing symptoms by inhalation of corticosteroids and β-2 adrenergic agonists. Recently, mice fed SCFAs showed the protective effects of SCFAs against allergic airway inflammation (122, 173). The inflow of eosinophils into the lung parenchyma is the signature feature of the most common allergic asthma.

During allergic inflammation, IL-5, IL-13, and granulocyte macrophage colony-stimulating factor (GM-CSF) secreted by Th2 and ILC2 cells promote eosinophil survival (174, 175). Activated eosinophils are major sources of cytokines, growth factors, and cytotoxic granulocytes (such as eosinophil-derived neurotoxins and major basic proteins) that can cause tissue damage and airway remodeling when released (176, 177). Recent *in vitro* studies using human peripheral blood eosinophils have shown that C3 and C4 inhibit eosinophils from adhering to endothelial cells in response to CCL24 flow, and C4 inhibits eosinophil migration and promotes eosinophil apoptosis (128). Surprisingly, these effects may be independent of GPR43 or GPR41 receptors but depend on the flow of these SCFAs into eosinophils *via* monocarboxylate transporters (128). In addition, we found that the biological effects of SCFAs on eosinophils are consistent with epigenetic regulation of gene expression by class IIa DAC, suggesting that these effects of SCFAs on eosinophils may be mediated by HDAC (128, 177). However, a previous study showed that diet-induced C3 prevents airway inflammation, resulting in decreased eosinophilic infiltration in lung tissue and decreased concentrations of the cytokines IL-4, IL-5, and IL-17A. This effect requires the participation of GPR41 but not GPR43 and is attributable to impaired DC activation (178, 179). Therefore, whether the effect of SCFAs on eosinophils depends on GPR43 or GPR41 receptors needs to be further investigated.

ILCs function to coordinate immunity, inflammation and tissue repair and can be present on the mucosal surface of the lung and drive allergic airway inflammatory responses (180, 181). ILC2s are of great concern in the context of asthma and allergic diseases because they promote Th2 immunity. Systemic and local administration of C4 attenuates ILC2-driven airway hyperresponsiveness and eosinophil inflammation. C4 can regulate the expression of ILC2s and inhibit their proliferation at the transcriptional level. C4 inhibits the proliferation of ILC2s and the production of the cytokines IL-5 and IL-13 by inhibiting the activity of HDAC without affecting cell viability and without being mediated by the activation of GPR41 or GPR43 (125, 182). C2 may increase the acetylation of the Foxp3 promoter through the inhibition of HDAC9, thus inhibiting the occurrence of allergic asthma (183). In addition, SCFAs can also affect allergic airway inflammation by affecting lung airway mast cells, Treg cells, Th9 cells, DCs, etc. (166, 173, 184–187). There are multiple mechanisms for the beneficial effects of SCFAs on the human airway immune inflammatory response, and further well-controlled long-term intervention studies are needed to confirm the beneficial effects of SCFAs in airway immune inflammatory diseases.

### Colitis

High dietary fiber intake and increased SCFA levels play an important role in protecting colon immune barrier function and in the colonic secretion of anti-inflammatory factors. SCFA administration can improve the symptoms of various types of colitis and reduce the probability of human colitis. Chronic intestinal inflammation can increase the risk of colon cancer. The concentration of SCFAs in stool is closely related to the incidence of colitis and colon cancer. SCFAs can reduce the risk of chronic colitis developing into colon cancer and promote the apoptosis of cancer cells to play an antitumor role (188). Studies have shown that the number of butyrate-producing bacteria in colon cancer patients is significantly reduced, and the expression of receptors GPR43 and GPR109A is also reduced considerably, indicating that SCFAs have a protective effect on colitis and colon cancer (189). The protective effect of SCFAs on colitis has been discussed extensively. SCFAs can regulate colon inflammation through innate immunity and antigen-specific adaptive immunity. As previously discussed, SCFAs can mediate a natural immune inflammatory response by inhibiting HDAC activity *via* GPR receptors. SCFAs can also affect intestinal IL-10 production and IgA secretion through multiple mechanisms. SCFAs generally show anti-inflammatory effects in the colon depending on the concentration and the immunological environment.

### Osteoporosis

Osteohomeostasis is maintained through coordination between the bone formation process managed by osteoblasts and bone resorption managed by osteoclasts. Probiotics prevent bone loss, promote bone formation and increase bone volume in mice (190–192). Previous studies have shown that SCFAs can indirectly stimulate bone formation. C4 increases the proportion of CD4+/CD8+ T cells and the number of Treg cells in the bone marrow. Treg cells activate NFAT and SMAD signal transduction, which results in indirect induction of Wnt10b production in CD8+ T cells and thus indirect stimulation of bone formation (193). A recent study showed that C3 and C4 directly upregulate osteoblast differentiation. Alkaline phosphatase (ALP) activity is a marker of osteogenic differentiation of mouse embryonic osteoblast progenitor cells (MC3T3-E1 cells). C2 and C3 increase the activity of ALP, and C2 increases the expression of ALP mRNA; however, C4 does not affect the activity of ALP or the expression of ALP mRNA (194). Osteopathic (OPN) is a highly phosphorylated and glycosylated salivary protein that is expressed in osteoblasts and osteoclasts (195). C2, C3 and C4 increase the expression of OPN in MC3T3-E1 cells (194). SCFAs can inhibit osteoclast differentiation (161). The differentiation of precursor cells into mature osteoclasts depends on oxidative phosphorylation, and the bone resorption of mature osteoclasts depends on glycolysis (196, 197). By inducing the metabolic recoding of osteoclasts, C3 and C4 weaken the oxidative phosphorylation of precursor cells through a process dependent on mature osteoclasts, enhance glycolysis, induce a stress response, and prevent osteoclast differentiation. Moreover, C3 and C4 downregulate the expression of the osteoclast genes TRAF6 and NFATC1 to inhibit osteoclast differentiation (192).

## Conclusion

Over the past few decades, by sequencing and analyzing different types of human gut microbiota and constructing their metabolic processes, researchers have recognized the important roles of microbial metabolites in health and disease mediated through microbe-host interactions. As one of the most important metabolites of intestinal microorganisms, SCFAs have been shown to regulate host physiology and health through innate and adaptive immunity. For example, SCFAs can affect the inflammatory response of the central nervous system and affect bone diseases (198–204). In addition, it can regulate rheumatic diseases, osteoarthritis, hepatitis, vasculitis and so on (169, 205–209). Although the current review has limitations, it is challenging to decipher all the complexities of the effects of intestinal SCFAs on immune metabolism. This calls for further exploration of the relationship between SCFA pairs and the immune system, which is critical for identifying treatment options based on SCFAs.

## Author contributions

All authors listed have made a substantial, direct, and intellectual contribution to the work and approved it for publication.
